# Degree of joint risk factor control and hazard of mortality in diabetes patients: a matched cohort study in UK Biobank

**DOI:** 10.1186/s12916-024-03288-0

**Published:** 2024-03-07

**Authors:** Jian Zhou, Xuan Wang, Rui Tang, Minghao Kou, Hao Ma, Xiang Li, Yoriko Heianza, Vivian Fonseca, Lu Qi

**Affiliations:** 1 Department of Epidemiology, Tulane University School of Public Health and Tropical Medicine, 1440 Canal Street, Suite 1724, New Orleans, LA 70112 USA; 2 Department of Orthopedics, The Second Xiangya Hospital of Central South University, Changsha, China; 3 Section of Endocrinology and Metabolism, Tulane University School of Medicine, New Orleans, LA USA; 4 Southeast Louisiana Veterans Health Care System, New Orleans, LA USA; 5 Department of Nutrition, Harvard T.H. Chan School of Public Health, Boston, MA USA

**Keywords:** Risk factor control, Mortality, Relative importance, UK Biobank

## Abstract

**Background:**

Diabetes patients are at higher risk for mortality than the general population; however, little is known about whether the excess mortality risk associated with diabetes could be mitigated or nullified via controlling for risk factors.

**Methods:**

We included 18,535 diabetes patients and 91,745 matched individuals without diabetes without baseline cancer or cardiovascular disease (CVD), followed up from 2006 to 2021. The main exposure was the number of optimized risk factors including glycated hemoglobin < 53 mmol/mole, systolic blood pressure < 140 mmHg and diastolic blood pressure < 90 mmHg, no albuminuria, non-current smoking and low-density lipoprotein cholesterol (LDL-C) < 2.5 mmol/L. We used Cox proportional hazards models to explore the association of the degree of risk factor control with all-cause mortality, cancer mortality, CVD mortality and other mortality.

**Results:**

Each additional risk factor control was associated with a 16, 10, 21 and 15% lower risk of all-cause mortality, cancer mortality, CVD mortality and other mortality, respectively. Optimal risk factors control (controlling 5 risk factors) was associated with a 50% (HR 0.50, 95% CI 0.41–0.62), 74% (HR 0.26, 95% CI 0.16–0.43) and 38% (HR 0.62, 95% CI 0.44–0.87) lower risk of all-cause mortality, CVD mortality and other mortality, respectively. Diabetes patients with 4, 3 and 5 or more controlled risk factors, respectively, showed no excess risk of all-cause mortality, cancer mortality and CVD mortality compared to matched non-diabetes patients.

**Conclusions:**

The results from this study indicate that optimal risk factor control may eliminate diabetes-related excess risk of all-cause mortality, CVD mortality and other mortality.

**Supplementary Information:**

The online version contains supplementary material available at 10.1186/s12916-024-03288-0.

## Background

Diabetes is a metabolic disorder characterized by hyperglycemia [1]. The worldwide impact of diabetes has significantly escalated over the previous two decades, and it is projected to affect over 700 million adults by the year 2045 [2]. Individuals with diabetes carry a 2 to 4 times higher risk of death than the general population [3].

Recently, we observed a significant association between degree of risk factor control and risk of CVD in diabetes patients [4]. Findings from prior studies have shown that controlling of risk factors such as elevated glycated hemoglobin, smoking and elevated blood pressure might be beneficial in preventing diabetes-related complications as well as death [5–7]. However, limited studies investigated the association of degree of joint risk factor control with all-cause and cause-specific mortality in diabetes patients, and very few studies have investigated whether the excess mortality risk associated with diabetes could be mitigated or nullified via controlling for risk factors including glycated hemoglobin, LDL-C, albuminuria, smoking and blood pressure. It is also unclear what degree of joint risk factor control could eliminate the diabetes-related excess risk of mortality.

The objective of this study was to scrutinize the associations of the degree of risk factors control with risks of all-cause mortality, cancer mortality, CVD mortality and other mortality in diabetes patients. We also compared diabetes patients with matched individuals without diabetes to determine whether the excess mortality risk associated with diabetes could be mitigated or nullified via controlling for risk factors. Furthermore, we evaluated the relative importance of these five risk factors in comparison to other lifestyle-related risk factors in relation to mortality.

## Methods

### Study population

The UK Biobank collected data from over half a million participants between the ages of 40 and 70 from 2006 to 2010. Each participant supplied comprehensive self-reported data at the start of the study through touchscreen questionnaires or via oral interviews at UK Biobank’s 27 evaluation centers. The details for design of UK Biobank have been described elsewhere [8]. The study conducted by the UK Biobank received approval from the National Health and Social Care Information Management Board, the North West Multicenter Research Ethics Committee (11/NW/0382), and Tulane University’s Institutional Review Board (2018–1872).

### Selection of participants

The participants with diabetes at baseline were defined based on UK Biobank algorithms by Eastwood et al. through hospital inpatient records, self-reported medical history and medication, which is a reliable measurement with 96% accuracy [9]. Moreover, the glycated hemoglobin A1c (HbA1c) ≥ 48 mmol/mol (6.5%) was also used to identify diabetes at baseline (Additional file 1: Table S1). A total of 502,505 participants were recruited from UK Biobank. After excluding 42,420 participants diagnosed with cancer and 29,013 participants diagnosed with CVD at baseline, 22,663 participants were diagnosed with diabetes. In total, 4119 diabetes patients without data for glycated hemoglobin, LDL-C, albuminuria, smoking or blood pressure were excluded and 9 unmatched diabetes patients were removed. A total of 18,535 diabetes patients were included and randomly matched for age, sex, and assessment center with 91,745 matched individuals without diabetes in this study (Additional file 1: Figure S1).

### Definition of risk factors control

According to previous guidelines and studies [10–13], we defined diabetes patients with different numbers of traditional risk factor control using five risk factors including glycated hemoglobin, LDL-C, albuminuria, smoking and blood pressure. Five risk factors including glycated hemoglobin, LDL-C, albuminuria, smoking and blood pressure were evaluated in our study (Additional file 1: Table S2) [10, 14]. (1) Glycated hemoglobin was quantified in a plasma sample gathered at baseline through high-performance liquid chromatography on a Bio-Rad VARIANT II Turbo (Bio-Rad Laboratories, Inc). Glycated hemoglobin control was established as glycated hemoglobin being less than 53 mmol/mol. (2) Serum LDL-C were analyzed using the enzymatic selective protection technique (Beckman Coulter UK, Ltd). We recognized lipid control as LDL-C being below 2.5 mmol/L [10]. (3) Urinary microalbumin was ascertained using immunoturbidimetric assays (Randox Bioscience) with a detection threshold of 6.7 mg/L. Urine creatinine was analyzed by enzymatic methods (Beckman Coulter UK, Ltd). For those participants who displayed detectable microalbumin levels, the urinary albumin-to-creatinine ratio (uACR) was calculated by microalbumin and creatinine measurements. In order to involve the maximum number of participants, for those with undetectable microalbumin levels, the uACR was estimated by pairing an assumed microalbumin level (6.7 mg/L) and creatinine, with any uACR equal to or above 3 mg/mmol recorded as missing values. Albuminuria was identified as a uACR equal to or above 3 mg/mmol, while its absence was considered controlled albuminuria. (4) Information about smoking status was gathered via a touchscreen questionnaire, comprising categories for never, past and current smoking. Those not currently smoking were classified as having controlled smoking. (5) A trained nurse conducted dual measurements of blood pressure using either an electronic blood pressure device (Omron 705 IT, OMRON Healthcare Europe B.V.), or, when necessary, a mercury sphygmomanometer was employed. The average systolic and diastolic pressures were subsequently computed as the mean of these readings. We established blood pressure control as systolic pressure less than 140 mmHg and diastolic less than 90 mmHg. Given the relatively small number of participants with 0 or 1 risk factor control, participants with 0 or 1 risk factor control were combined into risk factor control ≤ 1 group.

### Outcomes

The outcomes include all-cause mortality, cancer mortality, CVD mortality and other mortality. The cause and date of death were procured through linking to the UK and Wales National Health Service (NHS) Information Centre’s Death Registry, and the Scottish NHS Central Registry’s Death Registry for participants residing in Scotland. Additional insights regarding these death registries can be obtained from http://content.digital.nhs.uk/services. The end of follow-up was established either as the baseline date to death or the cut-off date (November 27, 2021), depending on which came first. Death outcomes were classified in accordance with the ICD 10. Within this study’s scope, we examined mortality from all-cause mortality, cancer (coded as C00 to C97), CVD (coded as I00 to I99) and other-cause [15, 16].

### Other variables

Age, sex, ethnic background, Townsend deprivation index, education years, alcohol intake frequency and use of insulin were self-reported. Body mass index (BMI) was derived by dividing the weight in kilograms by the height in meters squared (kg/m^2^). Healthy diet score was created according to the consumption of vegetables, fruits, fishes, processed meats and unprocessed red meats (Additional file 1: Table S3). The details of this scoring system have been outlined in our prior research [17, 18]. Adhering to global health guidelines for physical activity, we categorized participants into two groups based on their cumulative weekly minutes of moderate physical activity. Each 1 min of rigorous physical activity was treated as equal to 2 min of moderate exercise. The groups were delineated as less than 150 min moderate exercise per week, and 150 min or more moderate exercise per week [19]. A high cholesterol condition was characterized by a self-reported history of elevated cholesterol levels or the usage of cholesterol-lowering drugs. The duration of diabetes was determined by the span from the date of diabetes diagnosis to the baseline for diabetes patients and was designated as zero for control subjects without diabetes [10, 14]. The detailed information of all included variables can be obtained on the UK Biobank website (www.ukbiobank.ac.uk).

In order to compare the relative importance of risk factors and various covariates, we estimated the relative importance of risk factors using the *coxphERR* package from Rstudio 4.1.2 [20]. These included glycated hemoglobin, LDL-C, albuminuria, smoking, blood pressure, Townsend index, lower education, obesity (defined as BMI ≥ 30 kg/m^2^), alcohol, healthy diet, physical activity, high cholesterol, diabetes duration and use of insulin, in forecasting mortality among diabetes patients.

### Statistical analysis

Continuous variables were expressed as mean ± standard deviation, while categorical variables are shown as counts and percentages. We performed Cox proportional hazard regression models to investigate the association between degree of risk factor control and the risk of all-cause mortality, cancer mortality, CVD mortality and other mortality among the diabetes patients. The proportionality of hazards was verified using Schoenfeld residuals and Kaplan–Meier methods, and all tests satisfied the pre-set criteria. The group with the lowest degree (≤ 1) of risk factor control served as the reference group. The basic model was adjusted for age (years) and sex (male or female). The multivariable model further accounted for ethnic background (white or others), Townsend deprivation index (continuous), education years (continuous), BMI (< 25, 25– < 30, ≥ 30 kg/m^2^), alcohol intake (< 3 or ≥ 3 times/week), healthy diet score (< 3 or ≥ 3), physical activity (< 150 min/week or ≥ 150 min/week), high cholesterol (yes or no), diabetes duration (< 5, 5– < 10 or ≥ 10 years) and use of insulin (yes or no). We employed the same Cox models when comparing diabetes patients with matched non-diabetes patients. Missing data for categorical covariates and continuous variables were addressed with a missing indicator category and mean values, respectively. The count and percentage of participants with missing covariates are detailed in Additional file 1: Table S4.

### Sensitivity analyses

Two sensitivity analyses were carried out to verify the robustness of the findings. We excluded participants who were dead within the first 2 years of the follow-up period. Next, we imputed missing data for all covariates using chained equations. Statistical analyses were performed using SAS version 9.4 (SAS Institute, Cary, NC) and R version 4.1.2 (www.r-project.org), and we interpreted a two-sided *P*-value of less than 0.05 as indicative of statistically significant disparities.

## Results

### Baseline characteristics of participants

The baseline characteristics of the diabetes patients and matched controls are shown in Table 1. Among 18,535 diabetes patients, 6.2%, 21.1%, 36.9%, 28.0% and 7.8% had ≤ 1, 2, 3, 4 and 5 risk factors under control, respectively. Additionally, we observed that diabetes patients with higher degree of risk factor control were older, were more likely to be female, had higher socioeconomic status, eat healthier, were physically activated and were medication users. Moreover, they tend to have lower BMI, shorter diabetes diagnosed duration and type 2 diabetes.

**Table 1:** Baseline characteristics of included participants

Characteristics	Non-diabetes patients (*n* = 91,745)	Degree of joint risk factor control (*n* = 18,535)				
		≤ 1 risk factor (*n* = 1141)	2 risk factors (*n* = 3917)	3 risk factors (*n* = 6831)	4 risk factors (*n* = 5198)	5 risk factors (*n* = 1448)
Age, years, mean (SD)	58.4 (7.5)	57.9 (7.5)	58.4 (7.5)	58.8 (7.4)	58.4 (7.7)	58.4 (7.6)
Female, *n* (%)	36,717 (40)	367 (32.2)	1428 (36.5)	2835 (41.5)	2185 (42)	555 (38.3)
Ethnic background, *n* (%)						
Other	7427 (8.1)	188 (16.5)	482 (12.3)	765 (11.2)	631 (12.1)	170 (11.7)
White	83,810 (91.4)	944 (82.7)	3414 (87.2)	6041 (88.4)	4540 (87.3)	1263 (87.2)
Townsend deprivation index, mean (SD)	− 1.5 (3)	0.3 (3.5)	− 0.4 (3.3)	− 0.7 (3.4)	− 0.7 (3.4)	− 0.6 (3.4)
BMI, kg/m, *n* (%)						
< 25	29,384 (32)	98 (8.6)	348 (8.9)	761 (11.1)	775 (14.9)	242 (16.7)
25– < 30	41,742 (45.5)	363 (31.8)	1234 (31.5)	2415 (35.4)	1892 (36.4)	514 (35.5)
≥ 30	20,107 (21.9)	668 (58.6)	2304 (58.8)	3611 (52.9)	2503 (48.2)	685 (47.3)
Alcohol intake, times/week, *n* (%)						
< 3	47,655 (51.9)	814 (71.3)	2676 (68.3)	4567 (66.9)	3573 (68.7)	1044 (72.1)
≥ 3	43,843 (47.8)	326 (28.6)	1234 (31.5)	2251 (33)	1619 (31.2)	403 (27.8)
Healthy diet score, *n* (%)						
< 3	30,609 (33.4)	457 (40.1)	1453 (37.1)	2301 (33.7)	1652 (31.8)	451 (31.2)
≥ 3	57,103 (62.2)	583 (51.1)	2188 (55.9)	4107 (60.1)	3244 (62.4)	920 (63.5)
Physical activity, min/week, *n* (%)						
< 150	22,417 (24.4)	289 (25.3)	1066 (27.2)	1708 (25)	1436 (27.6)	384 (26.5)
≥ 150	51,175 (55.8)	460 (40.3)	1653 (42.2)	3111 (45.5)	2334 (44.9)	695 (48)
Diabetes duration, years, *n* (%)						
< 5	91,745 (100)	261 (22.9)	1121 (28.6)	2564 (37.5)	2129 (41)	673 (46.5)
5– < 10	0 (0)	226 (19.8)	862 (22)	1479 (21.7)	1281 (24.6)	389 (26.9)
≥ 10	0 (0)	348 (30.5)	991 (25.3)	1582 (23.2)	1130 (21.7)	314 (21.7)
Diabetes medication use, *n* (%)						
None	0 (0)	439 (38.5)	1624 (41.5)	3097 (45.3)	2158 (41.5)	448 (30.9)
Only insulin	0 (0)	141 (12.4)	440 (11.2)	644 (9.4)	439 (8.5)	96 (6.6)
Only oral medication	0 (0)	429 (37.6)	1509 (38.5)	2616 (38.3)	2286 (44)	824 (56.9)
Insulin and oral medications	0 (0)	132 (11.6)	344 (8.8)	474 (6.9)	315 (6.1)	80 (5.5)
Antihypertensive medication, *n* (%)	17,302 (18.9)	600 (52.6)	2096 (53.5)	3609 (52.8)	2776 (53.4)	808 (55.8)
Cholesterol-lowering medication, *n* (%)	12,428 (13.6)	637 (55.8)	2218 (56.6)	3962 (58)	3522 (67.8)	1200 (82.9)
Number of medications, mean (SD)	2.1 (2.3)	4.7 (3.5)	4.8 (3.3)	4.8 (3.1)	5 (3.2)	5.4 (3)
Number of operations, mean (SD)	1.6 (1.4)	1.6 (1.4)	1.7 (1.6)	1.7 (1.6)	1.8 (1.6)	1.7 (1.6)
Diabetes type, *n* (%)						
Type 1 diabetes	0 (0)	130 (11.4)	323 (8.3)	492 (7.2)	326 (6.3)	70 (4.8)
Type 2 diabetes	0 (0)	1008 (88.3)	3540 (90.4)	5977 (87.5)	4487 (86.3)	1321 (91.2)
Obesity, *n* (%)						
No	71,126 (77.5)	461 (40.4)	1582 (40.4)	3176 (46.5)	2667 (51.3)	756 (52.2)
Yes	20,107 (21.9)	668 (58.6)	2304 (58.8)	3611 (52.9)	2503 (48.2)	685 (47.3)

### Degree of joint risk factor control and hazard of mortality among diabetes patients

During a median follow-up period of 12.2 years, 2245 documented deaths were observed among 18,535 diabetes patients, including 843 cancer-related deaths, 563 CVD-related deaths and 839 other-related deaths. In basic model adjusted for age and sex, higher degree of joint risk factor control was significantly associated with lower risks of mortality from all-cause, cancer, CVD and other (Table 2). In the multivariable model, each additional risk factor control was associated with a 16% lower risk of all-cause mortality (HR 0.84; 95% CI 0.80–0.87), a 10% lower risk of cancer mortality (HR 0.90; 95% CI 0.84–0.96), a 21% lower risk of CVD mortality (HR 0.79; 95% CI 0.73–0.86) and a 15% lower risk of other mortality (HR 0.85, 95% CI 0.80–0.91) (all *P* for trend < 0.05). We observed that the group with optimal risk factors control (5 risk factors) was related to the lowest risk of all-cause mortality (HR 0.50; 95% CI 0.41–0.62), CVD mortality (HR 0.26; 95% CI 0.16–0.43) and other mortality (HR 0.62, 95% CI 0.44–0.87) (Table 2). Additionally, a directed acyclic graph (DAG) was created to explain the relationship between the exposures, the outcome and the covariates (Additional file 1: Figure S2). We observed that demographic characteristics (age, sex and ethnic background) may act as confounders. Socioeconomic factors (Townsend deprivation index), lifestyle factors (BMI, alcohol intake, healthy diet score and physical activity), diabetes-related factors (diabetes duration, diabetes type and diabetes medication use) and treatment-related factors (antihypertensive medication, cholesterol-lowering medication, number of medications and number of operations) may act as confounders or mediators or both in the association between degree of joint risk factor control and hazard of mortality in diabetes patients.

**Table 2:** Hazard ratios and 95% confidence intervals for association of degree of joint risk factor control with all-cause mortality and cause-specific mortality in diabetes patients (*n* = 18,535)

Outcomes	≤ 1 risk factor (*n* = 1141)	2 risk factors (*n* = 3917)	3 risk factors (*n* = 6831)	4 risk factors (*n* = 5198)	5 risk factors (*n* = 1448)	Per 1 risk factor control	*P*-trend
All-cause mortality							
Event, *n* (%)	228 (19.98)	559 (14.27)	798 (11.68)	517 (9.95)	143 (9.88)		
Basic model	1.00 (Reference)	0.67 (0.57–0.78)	0.54 (0.46–0.62)	0.46 (0.39–0.53)	0.45 (0.37–0.55)	0.81 (0.78–0.85)	< 0.001
Multivariable model	1.00 (Reference)	0.70 (0.60–0.82)	0.59 (0.51–0.69)	0.51 (0.43–0.59)	0.50 (0.41–0.62)	0.84 (0.80–0.87)	< 0.001
Cancer mortality							
Event, *n* (%)	63 (5.52)	213 (5.44)	308 (4.51)	199 (3.83)	60 (4.14)		
Basic model	1.00 (Reference)	0.96 (0.73–1.28)	0.79 (0.60–1.03)	0.67 (0.51–0.89)	0.73 (0.51–1.04)	0.88 (0.82–0.94)	< 0.001
Multivariable model	1.00 (Reference)	0.96 (0.73–1.28)	0.82 (0.62–1.07)	0.70 (0.53–0.94)	0.77 (0.53–1.10)	0.90 (0.84–0.96)	0.002
CVD mortality							
Event, *n* (%)	78 (6.84)	138 (3.52)	186 (2.72)	139 (2.67)	22 (1.52)		
Basic model	1.00 (Reference)	0.50 (0.38–0.66)	0.38 (0.30–0.50)	0.38 (0.29–0.50)	0.21 (0.13–0.34)	0.75 (0.69–0.81)	< 0.001
Multivariable model	1.00 (Reference)	0.55 (0.42–0.73)	0.46 (0.35–0.60)	0.46 (0.35–0.61)	0.26 (0.16–0.43)	0.79 (0.73–0.86)	< 0.001
Other mortality							
Event, *n* (%)	87 (7.62)	208 (5.31)	304 (4.45)	179 (3.44)	61 (4.21)		
Basic model	1.00 (Reference)	0.68 (0.53–0.87)	0.56 (0.44–0.71)	0.44 (0.34–0.56)	0.54 (0.39–0.74)	0.82 (0.77–0.88)	< 0.001
Multivariable model	1.00 (Reference)	0.74 (0.58–0.95)	0.66 (0.52–0.84)	0.50 (0.39–0.65)	0.62 (0.44–0.87)	0.85 (0.80–0.91)	< 0.001

*CVD* Cardiovascular disease

Basic model: adjusted for age and sex

Multivariable model: adjusted for age, sex, ethnic background, Townsend deprivation index, BMI, alcohol intake, healthy diet score, physical activity, diabetes duration, diabetes medication use, antihypertensive medication, cholesterol-lowering medication, number of medications, number of operations and diabetes type

Degree of joint risk factor control and diabetes-related excess risk of mortality among diabetes patients compared with matched individuals without diabetes.

**Fig. 1:**
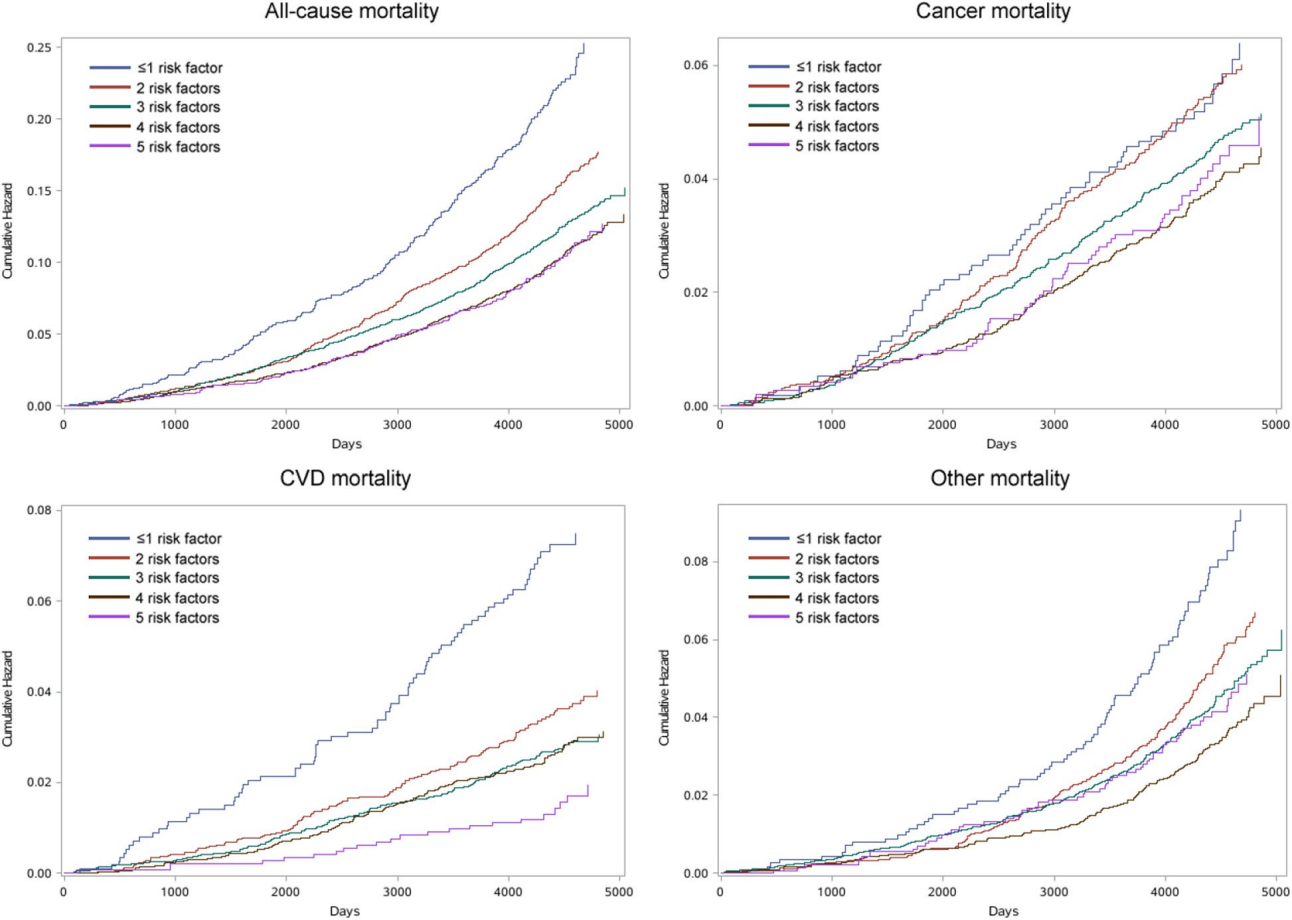
Cumulative hazard curves for the probability of all-cause mortality and causes-specific mortality in diabetes patients (*n* = 18,535) with varying degrees of joint risk factor control. CVD: cardiovascular disease

**Fig. 2:**
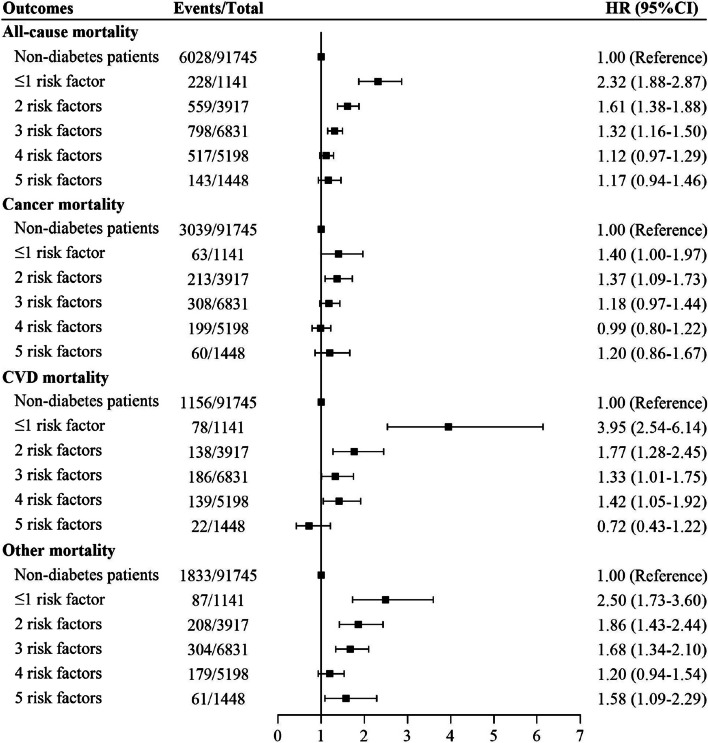
Associations of degree of joint risk factor control with risk for all-cause mortality and cause-specific mortality in diabetes patients (*n* = 18,535) compared with matched non-diabetes patients (*n* = 91,745) via multivariable model. CVD: cardiovascular disease. Multivariable model: adjusted for age, sex, ethnic background, Townsend deprivation index, BMI, alcohol intake, healthy diet score, physical activity, diabetes duration, diabetes medication use, antihypertensive medication, cholesterol-lowering medication, number of medications and number of operations

At the end of the follow-up period, the number of death was 6028 (6.6%) for non-diabetes patients, 228 (20.0%) for ≤ 1 risk factor control group, 559 (14.3) for 2 risk factor control group, 798 (11.7%) for 3 risk factor control group, 517 (10.0%) for 4 risk factor control group and 143 (9.9%) for 5 risk factor control group. Figure 1 illustrates the cumulative hazard curves representing the likelihood of mortality among diabetes patients with varying degrees of joint risk factor control. The respective cumulative mortality curves for participants with ≤ 1 risk factor control had the highest mortality rate throughout the follow-up period. To evaluate whether the diabetes-related mortality risk could be reduced by controlling for risk factors, we assessed mortality risk in relation to the degree of risk factor control among diabetes patients compared to their matched controls. During the follow-up, 6028 deaths were recorded in the matched control with 91,745 individuals without diabetes. As levels of risk factors decreased, lower risks were observed for all-cause mortality, cancer mortality and CVD mortality among diabetes patients, compared with non-diabetes patients (Fig. 2). Diabetes patients with 4, 3 and 5 or more risk factors under control exhibited no significant difference in risks of all-cause mortality (HR 1.17, 95% CI 0.94–1.46), cancer mortality (HR 1.18, 95% CI 0.97–1.44) and CVD mortality (HR 0.72, 95% CI 0.43–1.22) compared to matched controls. When the number of controlled risk factors increased from the lowest to the highest, the hazard ratio of all-cause mortality, cancer mortality and CVD mortality in diabetes patients decreased by 115, 20 and 323%, respectively, in comparison with individuals without diabetes (Fig. 2).

### Relative importance of the risk factors in relation to mortality risk in diabetes patients

**Fig. 3:**
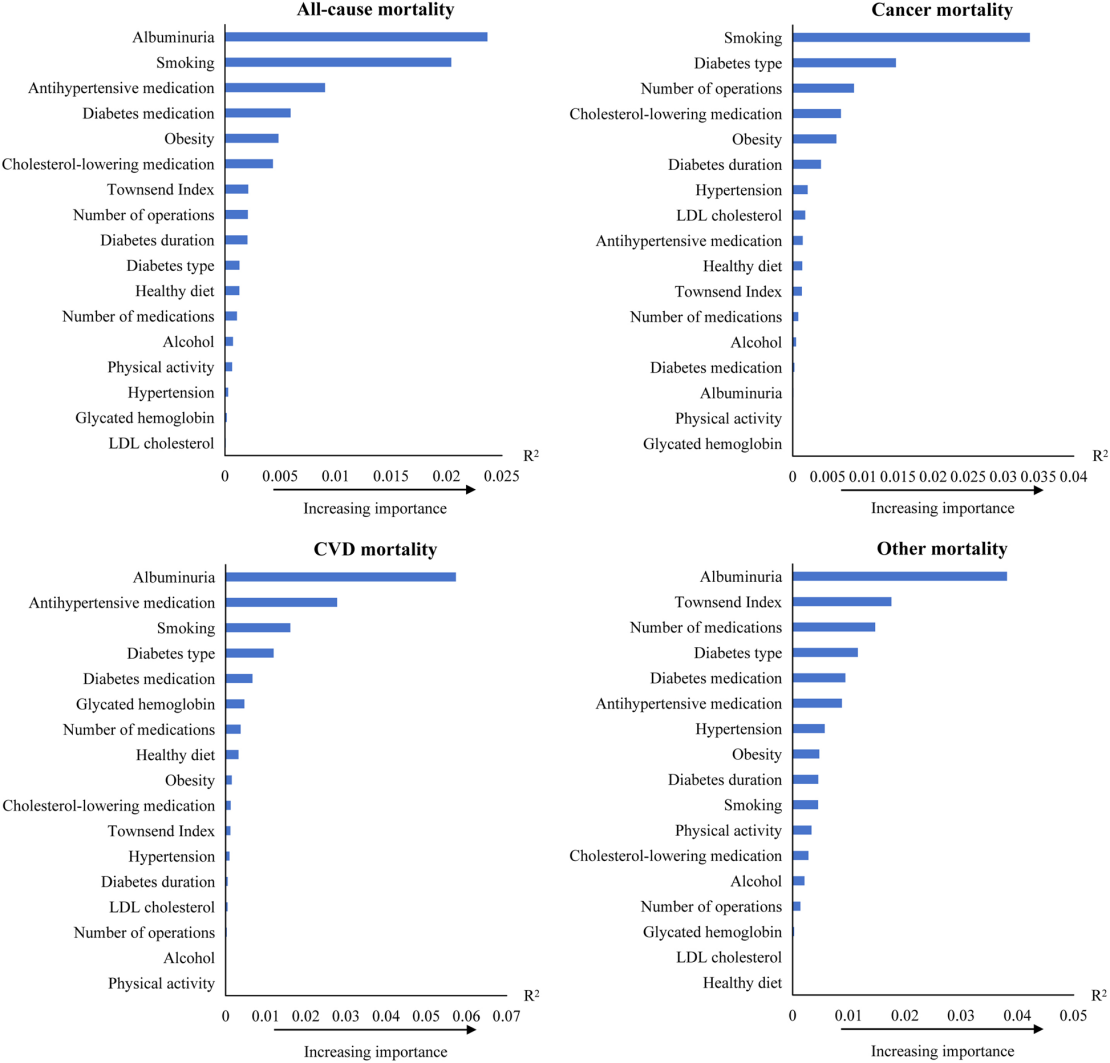
Relative importance of risk factors for all-cause mortality and cause-specific mortality in diabetes patients (*n* = 18,535). R^2^ was generated by developed applications for the multivariable model. CVD: cardiovascular disease. Multivariable model: adjusted for age, sex, ethnic background, Townsend deprivation index, BMI, alcohol intake, healthy diet score, physical activity, diabetes duration, diabetes medication use, antihypertensive medication, cholesterol-lowering medication, number of medications, number of operations and diabetes type

Figure 3 presents the risk factors associated with mortality. The three risk factors showing the strongest associations with all-cause mortality were albuminuria, smoking and use of antihypertensive medication in diabetes patients. Albuminuria showed the strongest associations with the risk of all-cause mortality, CVD mortality and other mortality. Smoking ranked as the second strongest predictor of all-cause mortality, the most potent predictor for cancer mortality, the third most potent for CVD mortality and the tenth most potent for other mortality.

### Sensitivity analysis

The correlations between degree of risk factor control and mortality remained largely unaltered when participants who were dead within the first 2 years of follow-up were excluded (Additional file 1: Table S5 & Additional file 1: Figure S3). Moreover, even after imputing all missing covariates through multiple imputation, the findings continued to be consistent (Additional file 1: Table S6 & Additional file 1: Figure S4).

## Discussion

In this prospective cohort study, we observed significant associations between the number of risk factor control and lower risks of all-cause and cause-specific mortality in diabetes patients. Each 1 additional risk factor control was associated with a 16, 10, 21 and 15% lower risk of all-cause mortality, cancer mortality, CVD mortality and other mortality, respectively. Optimal controlled for 5 risk factors was related to a 50, 74 and 38% lower risk of all-cause mortality, CVD mortality and other mortality, respectively. Diabetes patients with 4, 3 and 5 or more risk factors under control exhibited no significant difference in risks of all-cause mortality, cancer mortality and CVD mortality from matched non-diabetes patients.

Steno-2 trial indicated that multifactorial CVD risk factor intervention may reduce the risk of mortality in individuals with diabetes and microalbuminuria [21–23]. In this study, we present compelling evidence of the significant relationships between the degree of risk factors control with stepwise reduction of all-cause mortality, CVD mortality and other mortality in diabetes patients. The results of our study were supported by several previous studies [10, 24]. A cohort study using data from Swedish National Diabetes Register indicated that HR for all-cause mortality in T2D patients was 1.06 (95% CI 1.00–1.12) compared to non-T2D matched controls after optimal controlled for 5 risk factors [10]. Another cohort study presented that the excess risk of diabetes-related all-cause mortality may be substantially decreased with additional risk factor control [24].

In comparison to individuals without diabetes, there was a comparable progressive reduction in the excess risk of all-cause mortality, cancer mortality and CVD mortality with increasing degree of risk factor control. The HR for all-cause mortality, cancer mortality and CVD mortality decreased by 115, 20 and 323%, respectively, in diabetes patients in comparison with individuals without diabetes. Intriguingly, we found that by controlling 4, 3 and 5 or more risk factors, risk of all-cause mortality, cancer mortality and CVD mortality in diabetes patients became not different from those observed in non-diabetes patients. Similar results were observed in a previous study showing that optimal risk factor control could eliminate the all-cause mortality related to T2D [10]. Though the advantages of optimal risk factor control were reported previously [10, 11], our study revealed that merely 7.8% diabetes patients had five risk factors controlled, suggesting that the management of risk factors in diabetes patients could be considerably improved.

Additionally, we observed that controlling for relatively few risk factors in diabetes patients eliminated the risk of cancer mortality. Although the risk of cancer mortality was elevated in diabetes patients [25], the risk of cancer mortality in diabetes patients might be lower compared with the risk of CVD mortality and other mortality [26]. Moreover, although the diabetes-related excess risk from other mortality was not entirely eliminated after optimal risk factor control, the risks of other mortality among diabetes patients were constantly reduced with increasing degree of risk factor control, suggesting that joint risk factor control can still provide benefit for other mortality.

Diabetes is a chronic disease that affects multiple organs and systems. CVD and chronic kidney diseases (CKD) are the main complications of diabetes patients, leading to elevated risks of all-cause mortality and CVD mortality. Different risk factor controls may augment each other in diminishing disease risk via unique mechanisms and simultaneous control of multiple risk factors may be synergistic. Simultaneous control of glycated hemoglobin, blood pressure, and LDL-C can significantly reduce the risk of coronary artery disease and heart failure, thereby reducing the risk of mortality [21, 23]. Simultaneous control of blood pressure and albuminuria can reduce the risk of kidney failure [27, 28], which may reduce mortality in diabetes patients. At the same time, cardiorenal mechanisms may contribute to heart failure in T2D patients [10], suggesting the benefit of joint control of CVD and CKD risk factors. However, the underlying mechanisms contributing to the cumulative benefits of joint risk factor control in reducing mortality risk warrant further exploration.

In order to assess the relative importance of each risk factor, we conducted a risk factors analysis. Our study presented that albuminuria was the strongest predictor of all-cause mortality, CVD mortality and other mortality, which was different from previous a study showing that smoking was the strongest predictor of all-cause mortality in diabetes patients [10]. Although smoking was the strongest predictor of cancer mortality in our study, it ranked second, third and tenth in predicting all-cause mortality, CVD mortality and other mortality, respectively. One possible explanation for these differences could be the demographic and clinical characteristics of the populations studied. Additionally, differences in enrollment time and length of follow-up may also lead to differences in results, while further research is then needed to explore the reasons for the differences.

Previous studies indicated that albuminuria was an independent risk factor for mortality [29, 30]. The positive correlation of microalbuminuria with mortality in diabetes was consistent across all the published studies. Previous studies revealed that albuminuria is a strong predictor of adverse CVD outcomes in T2D patients [30] and microalbuminuria was predictive of CVD morbidity and mortality in T2D patients [31]. Additionally, use of antihypertensive medication and smoking were observed to be strong predictors for all-cause and CVD mortality, which was supported by previous study indicating that antihypertensive treatment was related to lower risk of mortality and cardiovascular morbidity in diabetes patients [32]. Furthermore, smoking and diabetes are both principal risk factors for all-cause and CVD mortality, and diabetes individuals who smoke may face an augmented risk of all-cause and CVD mortality. Additionally, smoking may exacerbate the damage of endothelium of blood vessels which was already compromised due to high blood sugar levels and promote arteriosclerosis [33]. Additionally, smoking can raise the levels of LDL-C in the blood and lower high-density lipoprotein cholesterol (HDL-C) levels [34], which might accelerate the progression of arteriosclerosis.

In the present study, five risk factors including glycated hemoglobin, LDL-C, albuminuria, smoking and blood pressure were selected to analyze the association of joint risk factor control and risk or mortality in diabetes patients. Lifestyle management is acknowledged as a core element in treating diabetes. Further study may add lifestyle factors including physical activity and healthy diet to observe the joint association of risk factor control and healthy lifestyle with risk or mortality.

### Strengths and limitations

This investigation bears several strengths, including its prospective design for a large cohort of diabetes patients, and extensive details on covariates. Nevertheless, some limitations warrant mention. First, alterations in risk factor variables during the follow-up period were not considered. Second, the majority of our study population are White Europeans, necessitating further research to extend the findings to other racial or ethnic groups. Third, due to the study’s observational design, we were constrained in deriving causal relationships. Lastly, participants in the UK Biobank might exhibit healthier behaviors, which could impose restrictions on the applicability of our findings.

## Conclusions

Our research indicated that higher degree of risk factor control may benefit diabetes patients in reduction of diabetes-related excess mortality risk; and optimal joint risk factor control may lower the risk toward the level of non-diabetes patients.

### Supplementary Information

**Additional file 1: Figures S1-S4.** **Figure S1.** [Flowchart of participants selected]. **Figure S2.** [Directed Acyclic Graph (DAG) indicating the relationships among exposures, outcomes, and included covariates in the analyses]. **Figure S3.** [Association of degree of joint risk factor control with all-cause mortality and cause-specific mortality in diabetes patients (*n*=18,406) compared with matched non-diabetes patients (*n*=91,396) after excluding participants who were dead during the first two years of follow-up via multivariable model]. **Figure S4.** [Association of degree of joint risk factor control with all-cause mortality and cause-specific mortality in diabetes patients (*n*=18,535) compared with matched non-diabetes patients (*n*=91,745) with all missing covariate data imputed using multiple imputation via multivariable model]. **Tables S1-S6.** **Table S1.** ablDefinitions of diabetes at baseline]. **Table S2.** TEvaluation of risk factors in the UK Biobank]. **Table S3.** TAssessment of healthy diet score in the UK Biobank]. **Table S4.** TThe numbers and percentages of participants with missing covariates]. **Table S5.** TAssociation of degree of joint risk factor control with all-cause mortality and cause-specific mortality in diabetes patients (*n*=18,406) after excluding participants who were dead during the first two years of follow-up via multivariable model]. **Table S6.** TAssociation of degree of joint risk factor control with all-cause mortality and cause-specific mortality in diabetes patients (*n*=18,535) with all missing covariate data imputed using multiple imputation via multivariable model].

## Data Availability

This study has been conducted using the UK Biobank Resource, approved project number 29256. The UK Biobank will make the source data available to all bona fide researchers for all types of health-related research that is in the public interest, without preferential or exclusive access for any persons. All researchers will be subject to the same application process and approval criteria as specified by UK Biobank. For more details on the access procedure, see the UK Biobank website: http://www.ukbiobank.ac.uk/register-apply.

## Abbreviations

BMI: Body mass index
CVD: Cardiovascular disease
HbA1c: Glycated hemoglobin A1c
LDL-C: Low-density lipoprotein cholesterol
NHS: National Health Service
uACR: Urinary albumin-to-creatinine ratio
