# Supplementary material for: Degree of joint risk factor control and hazard of mortality in diabetes patients: a matched cohort study in UK Biobank
Source: BMC Med. 2024 Mar 7;22:108. doi: 10.1186/s12916-024-03288-0 (PMC10921580; doi:10.1186/s12916-024-03288-0)
Supplement: Supplementary file 1 — Additional file 1: Figures S1-S4. Figure S1. [Flowchart of participants selected]. Figure S2. [Directed Acyclic Graph (DAG) indicating the relationships among exposures, outcomes, and included covariates in the analyses]. Figure S3. [Association of degree of joint risk factor control with all-cause mortality and cause-specific mortality in diabetes patients (n=18,406) compared with matched non-diabetes patients (n=91,396) after excluding participants who were dead during the first two years of follow-up via multivariable model]. Figure S4. [Association of degree of joint risk factor control with all-cause mortality and cause-specific mortality in diabetes patients (n=18,535) compared with matched non-diabetes patients (n=91,745) with all missing covariate data imputed using multiple imputation via multivariable model]. Tables S1-S6. Table S1. ablDefinitions of diabetes at baseline]. Table S2. TEvaluation of risk factors in the UK Biobank]. Table S3. TAssessment of healthy diet score in the UK Biobank]. Table S4. TThe numbers and percentages of participants with missing covariates]. Table S5. TAssociation of degree of joint risk factor control with all-cause mortality and cause-specific mortality in diabetes patients (n=18,406) after excluding participants who were dead during the first two years of follow-up via multivariable model]. Table S6. TAssociation of degree of joint risk factor control with all-cause mortality and cause-specific mortality in diabetes patients (n=18,535) with all missing covariate data imputed using multiple imputation via multivariable model]. [file 12916_2024_3288_MOESM1_ESM.docx]

**Additional file 1**

Figure S1. Flowchart of participants selected.

Figure S2. Directed Acyclic Graph (DAG) indicating the relationships among exposures, outcomes, and included covariates in the analyses.

Figure S3. Association of degree of joint risk factor control with all-cause mortality and cause-specific mortality in diabetes patients (n=18,406) compared with matched non-diabetes patients (n=91,396) after excluding participants who were dead during the first two years of follow-up via multivariable model.

Figure S4. Association of degree of joint risk factor control with all-cause mortality and cause-specific mortality in diabetes patients (n=18,535) compared with matched non-diabetes patients (n=91,745) with all missing covariate data imputed using multiple imputation via multivariable model.

Table S1. Definitions of diabetes at baseline.

Table S2. Evaluation of risk factors in the UK Biobank.

Table S3. Assessment of healthy diet score in the UK Biobank.

Table S4. The numbers and percentages of participants with missing covariates.

Table S5. Association of degree of joint risk factor control with all-cause mortality and cause-specific mortality in diabetes patients (n=18,406) after excluding participants who were dead during the first two years of follow-up via multivariable model.

Table S6. Association of degree of joint risk factor control with all-cause mortality and cause-specific mortality in diabetes patients (n=18,535) with all missing covariate data imputed using multiple imputation via multivariable model.

**
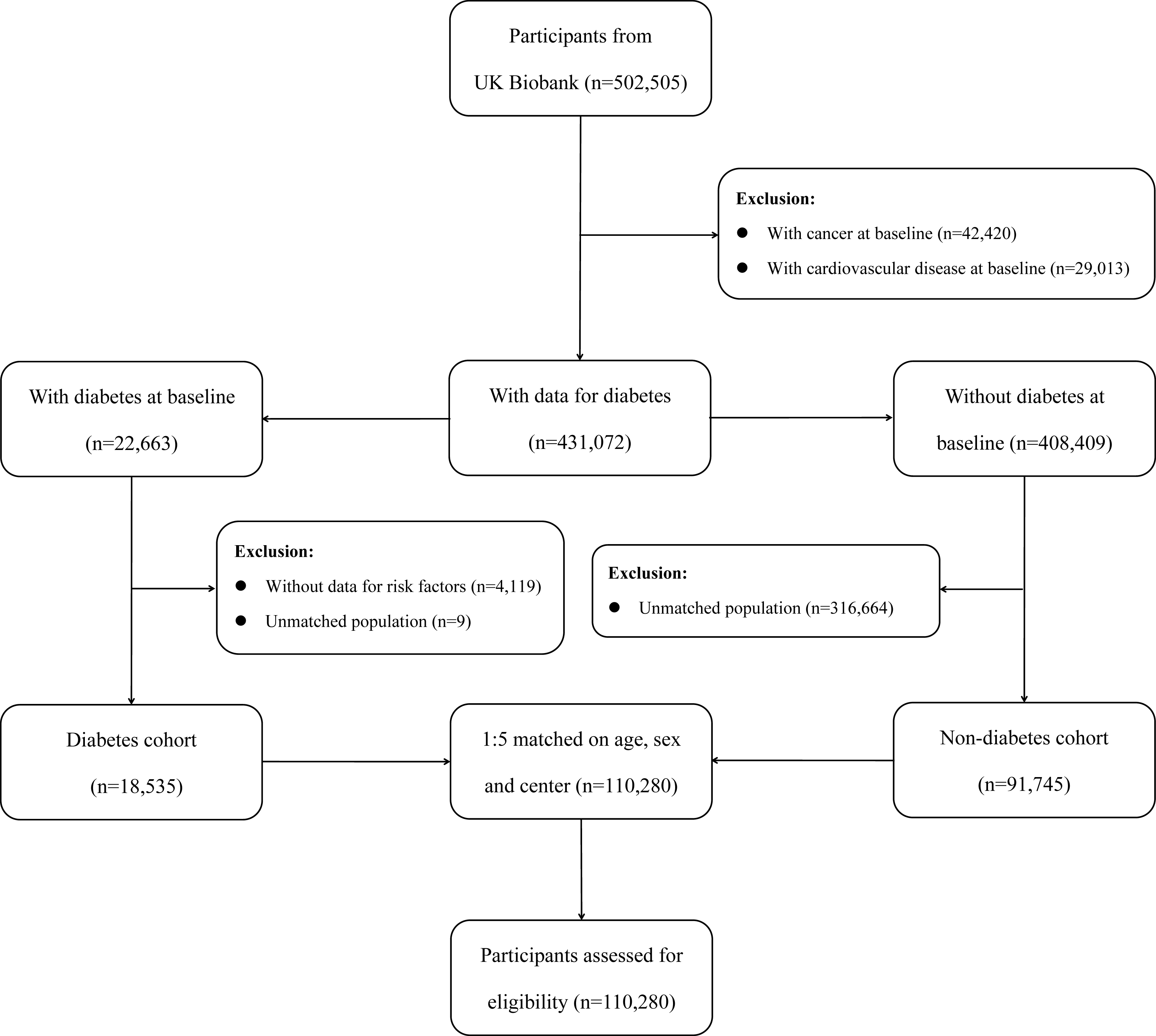
**

Figure S1. Flowchart of participants selected.


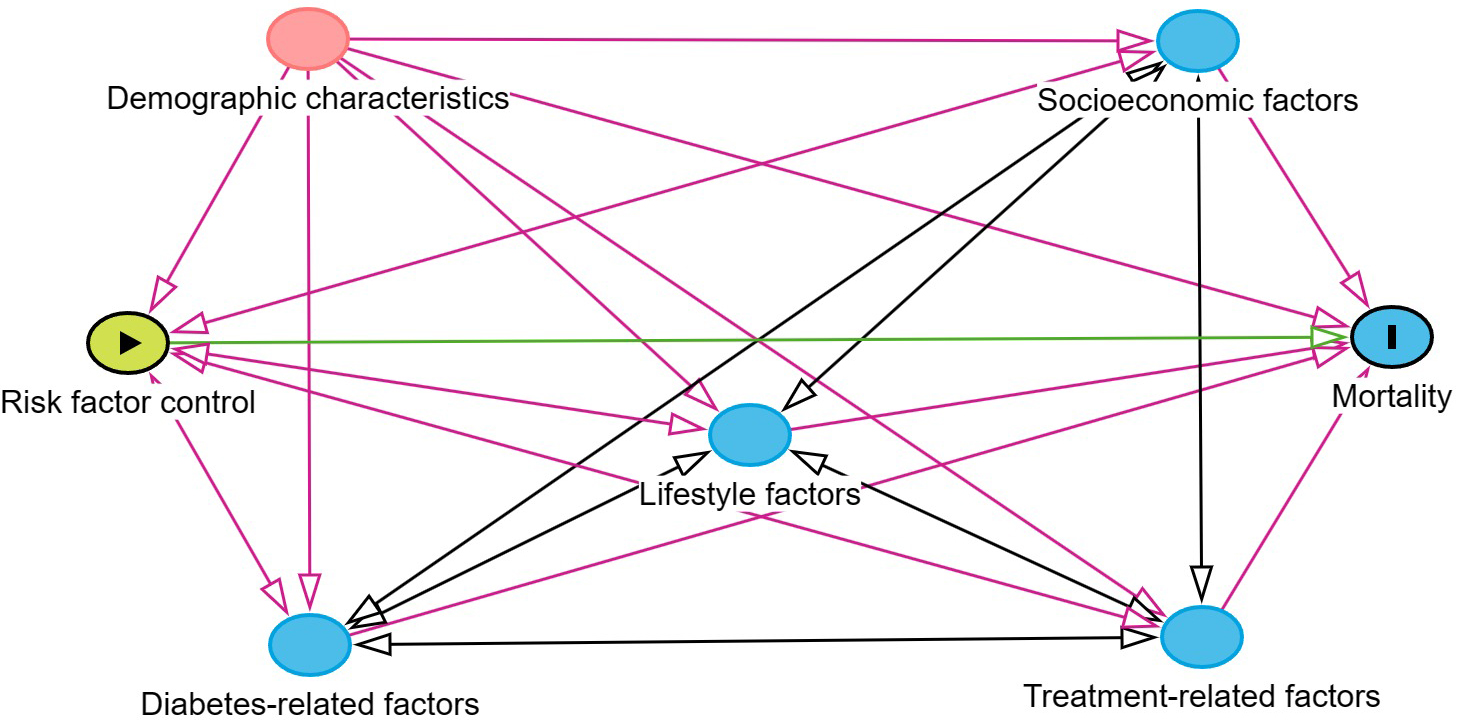


Figure S2. Directed Acyclic Graph (DAG) indicating the relationships among exposures, outcomes, and included covariates in the analyses.

Demographic characteristics (age, sex and ethnic background) may act as confounders. Socioeconomic factors (Townsend deprivation index), lifestyle factors (BMI, alcohol intake, healthy diet score and physical activity), diabetes-related factors (diabetes duration, diabetes type and diabetes medication use) and treatment-related factors (antihypertensive medication, cholesterol-lowering medication, number of medications and number of operations) may act as confounders or mediators or both in the association between degree of joint risk factor control and hazard of mortality in diabetes patients.

DAG was drawn using <http://www.dagitty.net/>


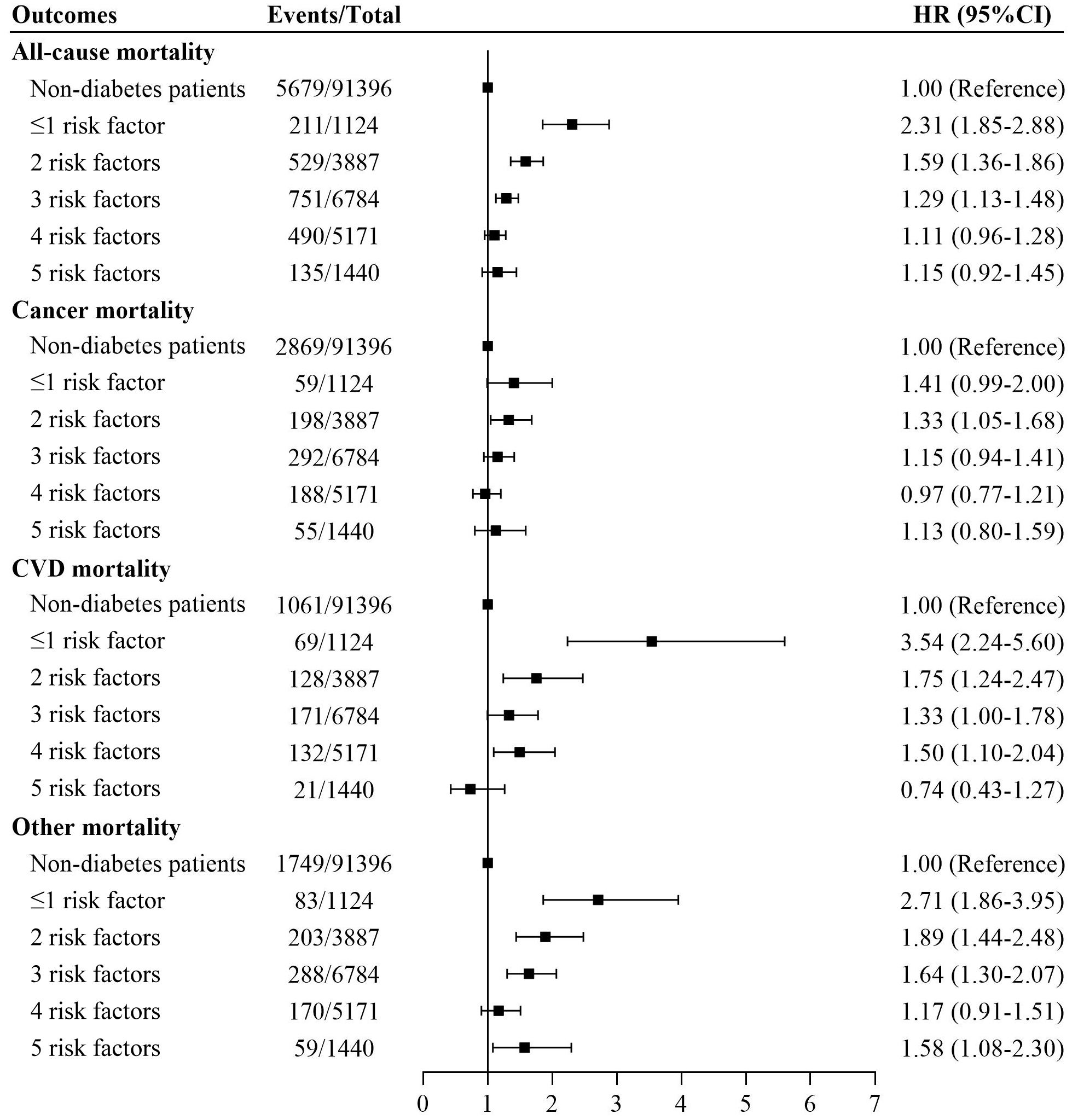


Figure S3. Association of degree of joint risk factor control with all-cause mortality and cause-specific mortality in diabetes patients (n=18,406) compared with matched non-diabetes patients (n=91,396) after excluding participants who were dead during the first two years of follow-up via multivariable model.

CVD: cardiovascular disease.

Multivariable model: adjusted for age, sex, ethnic background, Townsend deprivation index, BMI, alcohol intake, healthy diet score, physical activity, diabetes duration, diabetes medication use, antihypertensive medication, cholesterol-lowering medication, number of medications and number of operations.


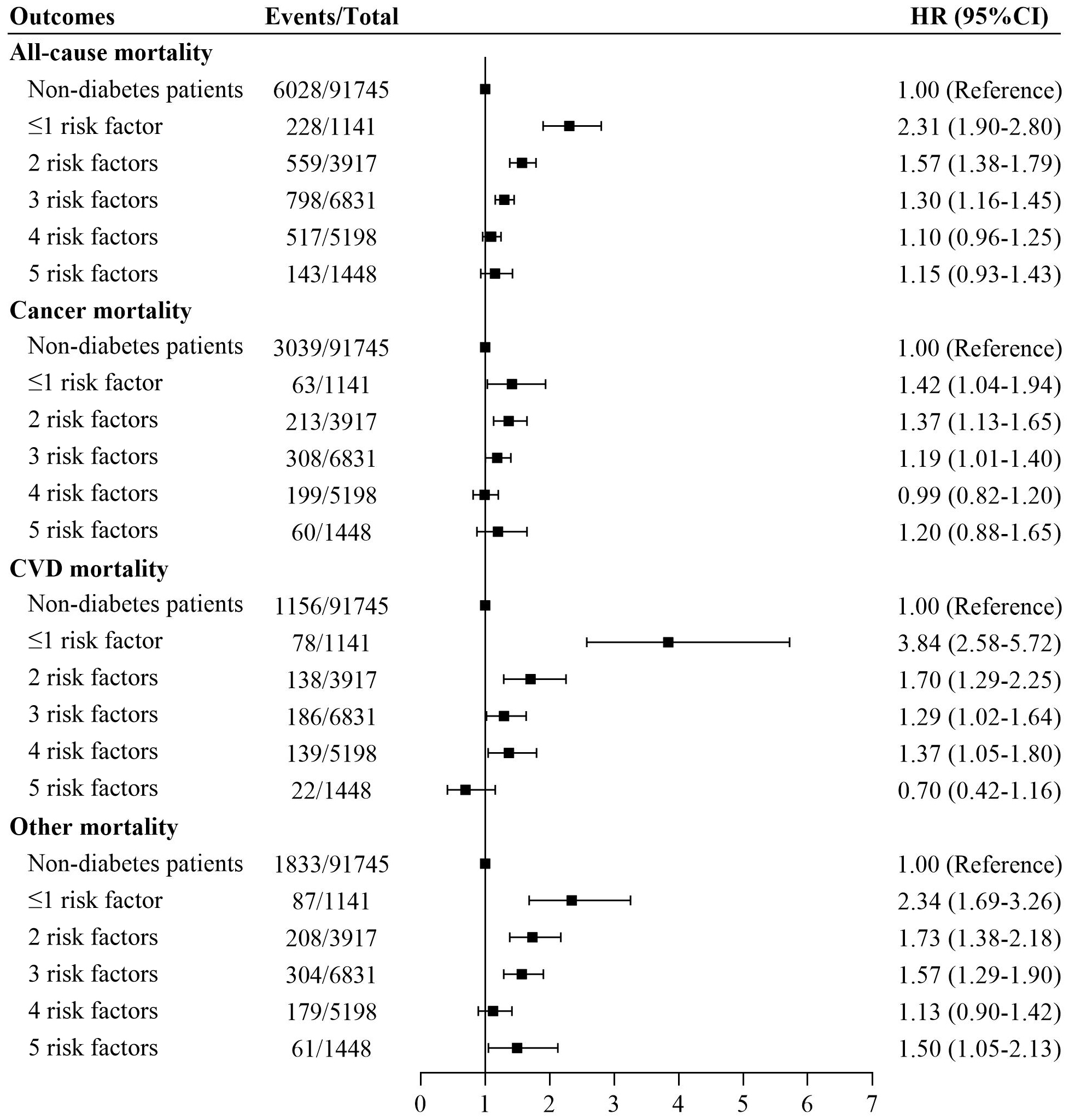
Figure S4. Association of degree of joint risk factor control with all-cause mortality and cause-specific mortality in diabetes patients (n=18,535) compared with matched non-diabetes patients (n=91,745) with all missing covariate data imputed using multiple imputation via multivariable model.

CVD: cardiovascular disease.

Multivariable model: adjusted for age, sex, ethnic background, Townsend deprivation index, BMI, alcohol intake, healthy diet score, physical activity, diabetes duration, diabetes medication use, antihypertensive medication, cholesterol-lowering medication, number of medications and number of operations.

Table S1. Definitions of diabetes at baseline.

|  | **UK Biobank ID** | **Code or definition** |
| --- | --- | --- |
| ICD 10 | 41270, 41280 | E10-E14 |
| ICD 9 | 41271, 41281 | 250 |
| First report | 130706, 130708, 130710, 130712, 130714 | Date E10-E14 first reported |
| Self report | 2443, 2976 | 1 |
|  | 20002, 20008, 20009 | 1220, 1222, 1223 |
| HbA1c | 30750 | HbA1c ≥ 48 mmol/mol |
| Insulin | 6153, 6177 | 3 |
|  | 20003 | 1140883066 |
| Other anti-diabetic medications | 20003 | 1140874646, 1140874718, 1141152590, 1140874674, 1140874706, 1140874744, 1141168660, 1141173882, 1140868902, 1141177600, 1141171646, 1140884600 |

Table S2. Evaluation of risk factors in the UK Biobank.

| **Risk factors** | **UK Biobank field ID** | **Description** | **Risk factor control** |
| --- | --- | --- | --- |
| Blood pressure | 93 | Systolic blood pressure, manual reading | Systolic blood pressure<140mmHg and Diastolic blood pressure <90mmHg |
|  | 94 | Diastolic blood pressure, manual reading |  |
|  | 4079 | Diastolic blood pressure, automated reading |  |
|  | 4080 | Systolic blood pressure, automated reading |  |
| Smoking | 20116 | Smoking status | Non-current smoker |
| Albuminuria | 30505 | Microalbumin in urine result flag | Microalbumin/Creatinine<3mg/mmol |
|  | 30510 | Creatinine (enzymatic) in urine |  |
| Glycated haemoglobin | 30750 | Glycated haemoglobin (HbA1c) | Glycated haemoglobin<53mmol/mol |
| LDL cholesterol | 30780 | LDL direct | LDL cholesterol<2.5mmol/L |

Table S3. Assessment of healthy diet score in the UK Biobank.

| **Diet** | **UK Biobank field ID** | **Description** | **Healthy diet score** |
| --- | --- | --- | --- |
| Vegetable | 1289 | Cooked vegetable intake | 1 for ≥4 tablespoons/day  0 for <4 tablespoons/day |
|  | 1299 | Salad/raw vegetable intake |  |
| Fruit | 1309 | Fresh fruit intake | 1 for ≥3 pieces/day  0 for <3 pieces/day |
|  | 1319 | Dried fruit intake |  |
| Fish | 1329 | Oily fish intake | 1 for ≥2 times/week  0 for <2 times/week |
|  | 1339 | Non-oily fish intake |  |
| Processed meat | 1349 | Processed meat intake | 1 for <2 times/week  0 for ≥2 times/week |
| Unprocessed red meat | 1369 | Beef intake | 1 for <2 times/week  0 for ≥2 times/week |
|  | 1379 | Lamb/mutton intake |  |
|  | 1389 | Pork intake |  |

Table S4. The numbers and percentages of participants with missing covariates.

| **Variable** | **Diabetes patients (n=18535)** | | **Diabetes patients and matched non-diabetes patients (n=110280)** | |
| --- | --- | --- | --- | --- |
|  | **N** | **%** | **N** | **%** |
| Physical activity | 5399 | 29.13 | 23552 | 21.36 |
| Diabetes duration | 3185 | 17.18 | 3185 | 2.89 |
| Healthy diet score | 1179 | 6.36 | 5212 | 4.73 |
| Antihypertensive medication | 210 | 1.13 | 1056 | 0.96 |
| Cholesterol-lowering medication | 210 | 1.13 | 1056 | 0.96 |
| BMI | 122 | 0.66 | 634 | 0.57 |
| Ethnic background | 97 | 0.52 | 605 | 0.55 |
| Alcohol intake | 28 | 0.15 | 275 | 0.25 |
| Number of medications | 27 | 0.15 | 138 | 0.13 |
| Number of medications | 0 | 0 | 159 | 0.14 |
| Number of operations | 0 | 0 | 159 | 0.14 |

Table S5. Association of degree of joint risk factor control with all-cause mortality and cause-specific mortality in diabetes patients (n=18,406) after excluding participants who were dead during the first two years of follow-up via multivariable model.

| **Outcomes** | **≤1 risk factor** | **2 risk factors** | **3 risk factors** | **4 risk factors** | **5 risk factors** | **Per 1 risk factor control** | ***P*-trend** |
| --- | --- | --- | --- | --- | --- | --- | --- |
| All-cause mortality | 1.00 (Reference) | 0.72 (0.61-0.84) | 0.60 (0.52-0.70) | 0.52 (0.44-0.61) | 0.51 (0.41-0.64) | 0.84 (0.81-0.88) | <0.001 |
| Cancer mortality | 1.00 (Reference) | 0.95 (0.71-1.27) | 0.82 (0.62-1.09) | 0.71 (0.53-0.96) | 0.75 (0.51-1.09) | 0.90 (0.84-0.97) | 0.003 |
| CVD mortality | 1.00 (Reference) | 0.56 (0.42-0.76) | 0.46 (0.35-0.62) | 0.49 (0.36-0.66) | 0.28 (0.17-0.45) | 0.80 (0.74-0.88) | <0.001 |
| Other mortality | 1.00 (Reference) | 0.74 (0.58-0.96) | 0.63 (0.49-0.81) | 0.49 (0.37-0.63) | 0.61 (0.43-0.86) | 0.84 (0.79-0.90) | <0.001 |

CVD: cardiovascular disease.

Multivariable model: adjusted for age, sex, ethnic background, Townsend deprivation index, BMI, alcohol intake, healthy diet score, physical activity, diabetes duration, diabetes medication use, antihypertensive medication, cholesterol-lowering medication, number of medications, number of operations and diabetes type.

Table S6. Association of degree of joint risk factor control with all-cause mortality and cause-specific mortality in diabetes patients (n=18,535) with all missing covariate data imputed using multiple imputation via multivariable model.

| **Outcomes** | **≤1 risk factor** | **2 risk factors** | **3 risk factors** | **4 risk factors** | **5 risk factors** | **Per 1 risk factor control** | ***P*-trend** |
| --- | --- | --- | --- | --- | --- | --- | --- |
| All-cause mortality | 1.00 (Reference) | 0.69 (0.59-0.81) | 0.58 (0.50-0.68) | 0.50 (0.42-0.58) | 0.49 (0.40-0.61) | 0.83 (0.80-0.87) | <0.001 |
| Cancer mortality | 1.00 (Reference) | 0.96 (0.72-1.27) | 0.81 (0.61-1.06) | 0.69 (0.52-0.92) | 0.75 (0.53-1.08) | 0.89 (0.83-0.95) | <0.001 |
| CVD mortality | 1.00 (Reference) | 0.54 (0.41-0.71) | 0.44 (0.34-0.58) | 0.45 (0.34-0.59) | 0.25 (0.16-0.41) | 0.78 (0.72-0.85) | <0.001 |
| Other mortality | 1.00 (Reference) | 0.72 (0.56-0.93) | 0.64 (0.50-0.82) | 0.49 (0.38-0.64) | 0.61 (0.44-0.85) | 0.85 (0.79-0.91) | <0.001 |

CVD: cardiovascular disease.

Multivariable model: adjusted for age, sex, ethnic background, Townsend deprivation index, BMI, alcohol intake, healthy diet score, physical activity, diabetes duration, diabetes medication use, antihypertensive medication, cholesterol-lowering medication, number of medications, number of operations and diabetes type.
